# Tetralogy of Fallot associated with macrocephaly-capillary malformation syndrome: a case report and review of the literature

**DOI:** 10.4076/1752-1947-3-9215

**Published:** 2009-09-08

**Authors:** Jesus E Dueñas-Arias, Eliakym Arámbula-Meraz, Luis O Frías-Castro, Rosalio Ramos-Payán, Jose A Quibrera-Matienzo, Fred Luque-Ortega, E Maribel Aguilar-Medina

**Affiliations:** 1 Departamento de Genética, Hospital Pediátrico de Sinaloa, Culiacán, Sinaloa, Mexico; 2 Posgrados en Biotecnología y Ciencias Biomédicas, Facultad de Ciencias Químico Biológicas, Universidad Autónoma de Sinaloa, Culiacán, Sinaloa, Mexico

## Abstract

**Introduction:**

Macrocephaly-capillary malformation syndrome is characterized by cutaneous vascular lesions, including cutis marmorata telangiectatica and hemangiomas, associated with congenital anomalies, including macrocephaly, macrosomia, asymmetry and mental retardation. In addition to these cardinal signs, several other clinical conditions have been reported in people with this condition. However, to the best of our knowledge, the presence of tetralogy of Fallot has not previously been reported in association with this syndrome.

**Case presentation:**

We present a case of a Mexican newborn girl with tetralogy of Fallot associated with macrocephaly-capillary malformation. We discuss the clinical treatment of the patient and its consequences.

**Conclusion:**

Since physiologic cutis marmorata is a common condition in newborns, the information provided in this report could be helpful in future cases in preventing severe clinical consequences or sudden death in patients with similar symptoms.

## Introduction

In 1975, Stephan *et al.* described 10 patients with cutaneous vascular anomalies, congenital macrocephaly and corporal asymmetry [1]. Until the detailed descriptions of new cases in 1997, however, the association among these cutaneous and congenital anomalies were recognized as a new syndrome called macrocephaly cutis marmorata telangiectatica congenita (M-CMTC) syndrome (Online Mendelian Inheritance in Man (OMIM) 602501) [2,3]. Recently, Toriello and Mulliken [4] suggested that the syndrome be renamed macrocephaly-capillary malformation (M-CM) because this term more accurately describes the cutaneous vascular anomalies. Approximately 100 cases of this condition have been reported, and men and women appear equally affected. The syndrome is characterized by cutaneous vascular malformations including cutis marmorata telangiectatica and hemangiomas associated with congenital anomalies such as macrosomia, asymmetry, macrocephaly and mental retardation. Other complications can be experienced by patients with this syndrome, including congenital cardiopathies, which can result in more severe complications. The presence of tetralogy of Fallot (ToF), however, has not been reported in any other patient. Here we described a newborn girl that presented with M-CM and ToF.

## Case report

An infant girl was born as the sixth child to 35-year-old unrelated Mexican parents without a remarkable family medical history. She was delivered by Cesarean section after only 38 weeks of gestation because polyhydramnios, hydrocephaly and heart defects were detected by prenatal ultrasound. Her Apgar scores were 7 at 1 minute and 8 at 5 minutes, her birth weight was 4.72 kg (>97th centile), and she was 53.5 cm in length and had a 44.5 cm head circumference.

**Figure 1:**
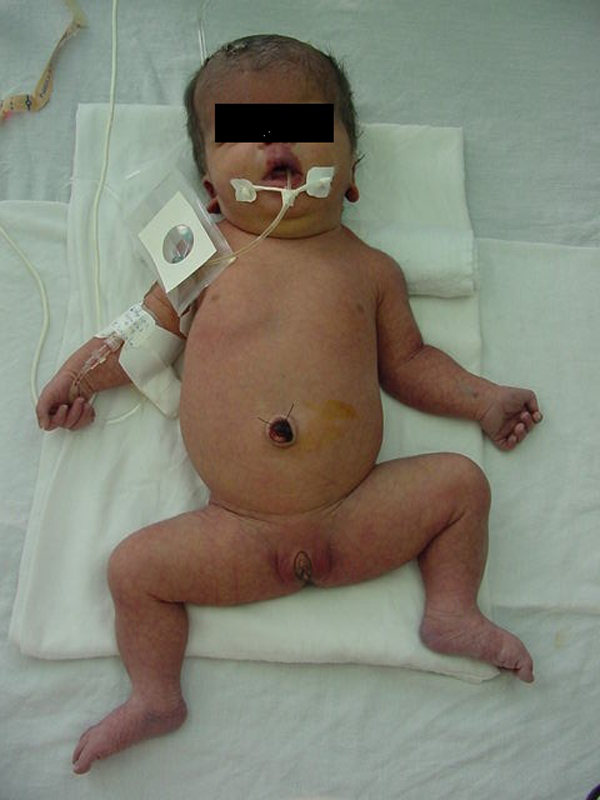
**Newborn girl with macrocephaly-capillary malformation**. Macrocephaly, macrosomia, cutis marmorata telangiectatica, capillary hemangioma of the lip and philtrum and hemihypertrophy can be observed.

The patient (Figure 1) was admitted to the Pediatric Hospital of Sinaloa, Mexico, because clinical examination confirmed the presence of hydrocephaly, cyanotic heart disease and several congenital anomalies such as macrosomia, cutis marmorata telangiectatica, macrocephaly (>97th centile), high forehead and frontal bossing, dolichocephaly, hypertelorism, capillary hemangioma of the lip and philtrum, nose with a low bridge and inverted nares, micrognathia, low-set ears, short neck, teletelia, hemihypertrophy of the left arm, diastasis recti, diastasis of the first and second toes of both feet, bilateral cutaneous syndactyly between second and third toes and a simian crease. A cytogenetic study using GTG banding of peripheral blood lymphocytes showed a normal 46,XX karyotype. At five months of age, her electrocardiogram showed normal sinus rhythm, right axis deviation (120º) and right ventricular hypertrophy.

A thoracic echo-Doppler ultrasound showed a ToF as indicated by the presence of a large ventricular septal defect, pulmonary stenosis, right ventricular hypertrophy and an overriding aorta. Axial computerized tomography imaging revealed hydrocephaly with ventricular dilatation, atrophy of the septum pellucidum and calcification in the left occipital region. Due to the hydrocephaly, ventriculoperitoneal shunting was necessary. The surgery was performed without complications. After 15 days, however, the infant suddenly died. Her parents did not allow an autopsy.

## Discussion

M-CM syndrome is characterized by macrocephaly, cutis marmorata telangiectatica, corporal asymmetry, macrosomia and hemangiomas of the lip and/or philtrum. The presence of these characteristics differentiates this syndrome from other related clinical entities such as the Klippel-Trenaunay-Weber, Sturge-Weber, CMTC (cutis marmorata telangiectatica congenita) and Bannayan-Riley-Rubalcaba syndromes. Other clinical manifestations can be found in M-CM, including congenital cardiopathies, which often result in more severe complications. To the best of our knowledge, however, the presence of ToF has not been reported in this syndrome.

Yano and Watanabe [5] reported three cases of patients with M-CMTC syndrome who suffered sudden deaths. In two of them, cardiac arrhythmia was observed suggesting a new severe clinical variant of the syndrome. Recently, Lapunzina *et al.*[6] described six new cases and reviewed another 69. They found four patients who presented with cardiac arrhythmia and died suddenly, and another five showing cardiac malformation. Additionally, Giuliano *et al.*[7] reported seven cases, one with severe cardiopathy resulting in death.

Approximately 100 cases of this condition have been reported, and men and women appear equally affected. The route of disease development remains a mystery. Some authors have suggested that the presence of a microdeletion is responsible for this syndrome [2,3,6].

Here we present the first documented case of M-CM with ToF in a Mexican newborn girl. In agreement with Yano and Watanabe [5], we consider that M-CM with severe congenital heart defects could increase the risk of life-threatening episodes or sudden death. Therefore, these complications should be taken into account in future cases.

## Conclusion

Since physiologic cutis marmorata is a common condition in newborns, the information provided in this report could be helpful in future cases in preventing severe clinical consequences or sudden death in patients with similar symptoms.

## List of abbreviations

CMTC: cutis marmorata telangiectatica congenital; M-CM: macrocephaly-capillary malformation; ToF: tetralogy of Fallot.

## Consent

Written informed consent was obtained from the mother of the newborn girl for publication of this case report and any accompanying images. A copy of the written consent is available for review by the Editor-in-Chief of this journal.

## Competing interests

The authors declare that they have no competing interests.

## Authors' contributions

JEDA provided genetic counselling to the parents. JAQM and LOFC gave cardiology, surgery and dermatology assistance. EMAM and EAM collected the data relevant to this case report. FLO and RPR conducted the data analysis, interpreted the investigation findings and revised the manuscript. EMAM, EAM and RPR performed the genetic studies and drafted the manuscript. All authors read and approved the final manuscript.

## References

[B1] Stephan MJ Hall BD Smith DW Cohen MM Jr Macrocephaly in association with unusual cutaneous angiomatosis J Pediatrics 1975 87 353 359 10.1016/S0022-3476(75)80634-2 170387

[B2] Clayton-Smith J Kerr B Brunner H Tranebjaerg L Magee A Hennekam RCM Mueller RF Brueton L Super M Steen-Johnsen J Donnai D Macrocephaly with cutis marmorata, haemangioma and syndactyly: a distinctive overgrowth syndrome Clin Dysmorph 1997 6 291 302 10.1097/00019605-199710000-00001 9354837

[B3] Moore CA Toriello HA Abuelo DN Bull MJ Curry CJR Hall BD Higgins JV Stevens CA Twersky S Weksberg R Dobys WB Macrocephaly-cutis marmorata telangiectatica congenita: a distinct disorder with developmental delay and connective tissue abnormality Am J Med Genet A 1997 70 67 73 10.1002/(SICI)1096-8628(19970502)70:1<67::AID-AJMG13>3.0.CO;2-V 9129744

[B4] Toriello HV Mulliken JB Accurately renaming macrocephaly-cutis marmorata telangiectatica congenita (M-CMTC) as macrocephaly-capillary malformation (M-CM) Am J Med Genet A 2007 143 3009 10.1002/ajmg.a.31971 17963258

[B5] Yano S Watanabe Y Association of arrhythmia and sudden death in macrocephaly-cutis marmorata telangiectatica congenita syndrome Am J Med Genet A 2001 102 149 152 10.1002/ajmg.1428 11477607

[B6] Lapunzina P Gairi A Delicado A Mori MA de Torres ML Goma A Navia M Lopez Pajares I Macrocephaly-cutis marmorata telangiectasia congenita: report of six new patients and a review Am J Med Genet A 2004 130 45 51 10.1002/ajmg.a.30235 15368495

[B7] Giuliano F David A Edery P Sigaudy S Bonneau D Cormier-Daire V Philip N Macrocephaly-cutis marmorata telangiectatica congenita: seven cases including two with unusual cerebral manifestations Am J Med Genet A 2004 126 99 103 10.1002/ajmg.a.20551 15039980

